# Triple Hernia

**Published:** 1861-08

**Authors:** 


must guard against exposure. Above all he must be removed at once from the influence of the miasmatic poison which, causing the trouble originally, is capable of maintaining its evil effects, and of neutralizing all attempts at cure. If medical interference be deemed necessary, you will fail if you resort to iodine or mercurials; but the agent, so specific in the intermittents,—*quinine*,—will be found most beneficial. This may be given in grain doses, three times a day, for two or three weeks, then intermit for a like space, and renew; this seems to be a necessary precaution against the action of the salt wearing out by long use.

This lad's occupation (that of a news-boy) keeps him in the open air a good deal, and the observance of the hints already given, are gradually and steadily improving his condition, so that now, aside from the somewhat aldermanic proportions of his abdomen, he is comfortable, and enjoys a very good degree of health.

II. *Triple Hernia.**—I show you here an interesting case presenting the unusual combination of three congenital hernias, of which two are scrotal, and one umbilical. Congenital hernias are the result of arrested development, in consequence of which the inguinal or umbilical canals remain open after birth, instead of closing at the proper period. The scrotal hernias, you see, are quite large, but easily reduced, and in nothing different from other tumors of the same kind. The umbilical rupture is small and soft. It is common to meet this congenital defect in any one of the three places which you see here occupied, but it is very rare to find the patient having them all at once.

Fortunately, although the canals in question occasionally remain open until after birth, yet the fœtal tendency to close them still continues for many months. Hence, in these cases we may almost always obtain a radical cure without any operative interference. All that is necessary is to keep the hernia constantly reduced, so that the presence of the intestine shall not prevent the contraction of the canal. By this simple

---

* Service, Prof. E. ANDREWS, Attending Surgeon.

treatment the tube, in from one to eight months, will close of itself, and put a final end to the difficulty.

In the umbilical hernia the treatment is not only simple in principle, but easy in practice. All that is required is a flat compress, made of sheet lead, or any other convenient material, and a band to keep it constantly in place. The nurse must be instructed to hold her finger upon the tumor whenever the compress is changed for purposes of cleanliness.

The inguinal hernia is more difficult to treat, because the infant is usually too small to be fitted by any ready made truss. If you have access to an ingenious mechanic you may order a truss made for the case; but, if not, you must construct one upon the principle of the compress and *T* bandage. The practical difficulty is that the child constantly soils the bandage and compress with its excrements. This may be met by having a sufficient supply of the dressings on hand, so that like the diapers, they can be changed and washed as many times a day as is necessary. Of course, the nurse must be instructed how to apply them, and where to make pressure with the fingers, for the purpose of keeping up the intestine, when she makes the change of bandages.

